# Breakfast and Energy Drink Consumption in Secondary School Children: Breakfast Omission, in Isolation or in Combination with Frequent Energy Drink Use, is Associated with Stress, Anxiety, and Depression Cross-Sectionally, but not at 6-Month Follow-Up

**DOI:** 10.3389/fpsyg.2016.00106

**Published:** 2016-02-09

**Authors:** Gareth Richards, Andrew P. Smith

**Affiliations:** Centre for Occupational and Health Psychology, School of Psychology, Cardiff UniversityCardiff, UK

**Keywords:** adolescent behavior, anxiety, breakfast, caffeine, depression, energy drinks, mental health, stress

## Abstract

A considerable amount of research suggests that breakfast omission and the frequent use of caffeinated energy drinks may be associated with undesirable effects, and particularly so in children and adolescents. The current paper presents cross-sectional and longitudinal data from the Cornish Academies Project to investigate the effects of consuming energy drinks and missing breakfast on stress, anxiety, and depression in a cohort of secondary school children from the South West of England. Questionnaires were administered at two time-points (spaced 6 months apart) to collect information relating to diet and lifestyle over the previous 6 months. Demographic and school data were acquired through the School Information Management System, and single-item measures of stress, anxiety, and depression were administered at the second time-point only. Associations between breakfast and energy drink consumption and stress, anxiety, and depression were investigated, and a multivariate approach was taken so that additional variance from diet, demography, and lifestyle could be controlled for statistically. Cross-sectional analyses showed that breakfast omission was consistently associated with negative outcomes, and that this was largely observed for both those who frequently consumed energy drinks and those who did not. However, cross-lag analyses showed that neither breakfast omission or energy drink consumption, alone or in combination, was predictive of stress, anxiety, or depression at 6-month follow-up. This suggests that associations between breakfast and mental health may be bi-directional rather than breakfast being the causal factor.

## Introduction

Energy drinks are highly caffeinated soft drinks that are purported to increase performance and endurance (Meadows-Oliver and Ryan-Krause, 2007). Concern has been raised due to such products sometimes being marketed as functional foods or dietary supplements, bypassing legislation regarding caffeine content (McCusker et al., 2006; Pomeranz, 2012), and therefore having the potential to put users at risk of intoxication (Reissig et al., 2009; Seifert et al., 2011). What is particularly alarming is the use of energy drinks by young consumers, with the Committee on Nutrition and the Council on Sports Medicine and Fitness (2011, p. 1182) claiming that “caffeine and other stimulant substances contained in energy drinks have no place in the diet of children and adolescents.”

As caffeine appears to be the main active ingredient in energy drinks (McLellan and Lieberman, 2012), it is important to consider its effects on mood and behavior. If consumed in moderation, it appears not to induce serious adverse health effects in adults (Nawrot et al., 2003) or children (Mandel, 2002; Higdon and Frei, 2006), though it has been advised for those highly sensitive not to consume >400 mg/d (Nawrot et al., 2003). Caffeine use does however appear to have important implications in the school environment. Its high consumption has, for instance, been associated with difficulty sleeping and morning tiredness (Orbeta et al., 2006), falling asleep at school (Calamaro et al., 2009), anger (Kristjánsson et al., 2011), violence and conduct disorder (Kristjánsson et al., 2013), and low academic attainment (James et al., 2011). When specifically investigating energy drinks it may therefore be beneficial to take caffeine consumption into account, otherwise the effects could be wrongly ascribed.

The frequency and amount of consumption of many food and drink products is known to be heavily inter-correlated (Wiles et al., 2009), and there is evidence to suggest that the effects of energy drinks may be best understood in combination with other aspects of diet. Richards et al. (2015a), for instance, reported a combined effect, in which energy drink consumption and breakfast omission were together predictive of the acute occurrence of detentions in secondary school children. Breakfast consumption is itself known to be associated with a number of cognitive benefits to children and adolescents (see Hoyland et al., 2009, for a review). Though some studies (e.g., Dickie and Bender, 1982; Cromer et al., 1990; López et al., 1993) have found no cognitive benefit of breakfast compared to its omission, others have reported a number of advantages, such as improvements in attention (Wesnes et al., 2003; Ingwersen et al., 2007), short-term memory (Smith et al., 1994; Vaisman et al., 1996; Benton and Parker, 1998; Wesnes et al., 2003), long-term memory (Chandler et al., 1995), creativity (Wyon et al., 1997), arithmetic (Powell et al., 1998), mood (Smith et al., 1999), and behavior (Bro et al., 1994). Due to such observations, the regular consumption of breakfast is generally considered to be of importance, and particularly so in relation to children and adolescents. However, it should also be noted that a recent review by Brown et al. (2013) found evidence to suggest that the scientific literature relating to the proposed effect of diet on obesity suffers from distortions caused by research lacking probative value, and biased research reporting. It may therefore be that similar biases exist in relation to the literature on associations between breakfast and other outcomes, such as cognitive performance, memory, and behavior.

The current study aimed to examine associations between missing breakfast and consuming energy drinks, and self-reported stress, anxiety and depression in secondary school children. A multivariate approach to analysis was adopted so that variance from other aspects of diet, demography, and lifestyle could be accounted for. Combined effects of energy drinks and breakfast were also investigated, and it was hypothesized that breakfast omission in combination with frequent energy drink use would be the strongest predictor of undesirable outcomes.

## Materials and Methods

### Participants

Data presented in the current paper came from the Cornish Academies Project, a longitudinal study of diet in secondary school children that consisted of two cross-sections. Data at Time 1 (T1; December 2012) were collected 6 months prior to Time 2 (T2; June 2013). However, as the stress, anxiety, and depression variables were only collected at T2, data from T1 are only used here when presenting cross-lag analyses. 3071 pupils were asked to take part in the research at T1, and 2610 (85%) agreed. At T2, the cohort consisted of 3323 pupils (Academy 1 *N* = 971, Academy 2 *N* = 1375, Academy 3 *N* = 977), 2307 of which completed the questionnaires, providing a response rate of 88.4% (slightly higher than the 77.8% reported at T1). The sample consisted of 48.5% males and 51.5% females, an age range of 11–17 (*M* = 13.6, *SD* = 1.49) was observed, and similar percentages of participants came from each year group of secondary education (Year 7 = 18.8%, Year 8 = 19.7%, Year 9 = 20.3%, Year 10 = 20.2%, Year 11 = 21%). A relatively high proportion of pupils (29.2%) had a special educational needs (SEN) status, 13.1% were eligible to receive free school meals (FSM), and almost all pupils were White (97.2%), spoke English as their first language (98.3%), and were not looked after by a non-parental guardian (99.4%).

### Apparatus/Materials

The Diet and Behaviour Scale (DABS; Richards et al., 2015b) was used as a measure of dietary intake of common foods and drinks. The questionnaire focuses on functional foods, and foods and drinks that may have effects on psychological outcomes (examples include breakfast, energy drinks, coffee, tea, cola, chewing gum, fruit, and vegetables). Eighteen questions assess the frequency of dietary intake on a five-point scale [1 = never, 2 = once a month, 3 = once or twice a week, 4 = most days (3–6), 5 = every day], and 11 items measure the amount of consumption. Through factor analyzing the whole scale, Richards et al. (2015b) found the measure to be associated with a four-factor structure labeled ‘Junk Food,’ ‘Caffeinated Soft Drinks/Gum,’ ‘Healthy Foods,’ and ‘Hot caffeinated Beverages.’ Subscales derived from the Junk Food factor (comprised of items measuring both the frequency and amount of consumption of chocolate and crisps, and items measuring the frequency of consumption of sweets and chips) and the Healthy Foods factor (comprised of items measuring both the frequency and amount of consumption of fruit and vegetables) were used as control variables in the current study.

Alongside the DABS, participants were administered a questionnaire to record the frequency by which they took part in exercise (mildly energetic, moderately energetic, and vigorous), on a four-point scale (1 = three times a week or more, 2 = once or twice a week, 3 = about once to three times a month, 4 = never/hardly ever). These three items were factor analyzed to produce a single factor solution (the details of which can be found in Richards et al., 2015b), which was used as a control variable in the current study in order to avoid unnecessarily compromising the statistical power of multivariate analyses. In addition, participants also reported the number of hours for which they normally slept each night.

Questions were administered to measure self-assessed mental health at T2 only. Participants were asked to specify how frequently they had felt stressed, anxious, and depressed during the previous 6 months. Answers to these questions were provided on a five-point scale (1 = not at all, 2 = rarely, 3 = sometimes, 4 = frequently, 5 = very frequently). Single items were used because they have been shown to be valid and reliable, and are less time-consuming to administer than multi-item measures (Williams and Smith, 2012). These particular items were taken from the Wellbeing Process Questionnaire (WPQ; Williams, 2014), and have been validated against full-length scales.

### Design and Procedure

Teachers administered the questionnaires to pupils at their schools. Demographic information was then obtained through the School Information Management System and stored in a confidential database at Cardiff University. This information included sex, age, school attended, year group, SEN status, and the eligibility/ineligibility to receive FSM (the latter being used as an indication of socioeconomic status; Shuttleworth, 1995).

All questionnaire and demographic data were anonymized prior to being merged into a single database. Cardiff University’s School of Psychology Ethics Committee granted the study ethical clearance, informed consent was acquired from all participants (as well as from their parents), and the research was conducted in accordance with the Declaration of Helsinki.

### Statistical Analysis

Both the predictor variables and the outcomes were measured using Likert response format items. There has been considerable debate (Carifio and Perla, 2007) about whether these can be considered as continuous variables (multi-item scales may be considered continuous but single items usually are not) and a conservative approach conceptualizes the Likert response type single items as ordered categorical variables (Jamieson, 2004). The distribution across the categories also means that it is appropriate to reduce the problems of unequal or small sample sizes by dichotomizing the scores using median splits. This approach has been adopted in other studies from the Cornish Academies Project and was continued here.

The predictor variables (breakfast and energy drink consumption) and outcomes (stress, anxiety, and depression) were dichotomized, and relationships with dietary, demographic, and lifestyle covariates were investigated using Chi-square and between subjects *t*-tests. Cross-sectional relationships between the frequency of breakfast and energy drink consumption and stress, anxiety, and depression were then investigated using binary logistic regression analysis (using enter method), so that aspects of demography (sex, school attended, school year, presence/absence of a SEN status, eligibility/ineligibility to receive FSM) and lifestyle (sleep hours, exercise frequency, and school attendance) could be controlled for statistically. In addition to this, as Richards and Smith (2015a) observed caffeine consumption to be associated with mental health outcomes in this same sample of secondary school children, its weekly intake from cola, tea, and coffee were entered as covariates. Caffeine consumed from energy drinks was also entered when the indicator variable was breakfast (though not when the indicator variable was energy drinks). To control for other aspects of diet, the DABS subscales for Junk Food and Healthy Foods were also used; however, the subscales for Caffeinated Soft Drinks/Gum and Hot Caffeinated Beverages were not entered due to them being comprised of caffeinated products (energy drinks and cola in the case of the former, tea, and coffee in the case of the latter).

In order to further examine the nature of the relationships observed, the frequency of breakfast and energy drink consumption variables were combined so that all four possible groupings of frequent/infrequent intake could be investigated in relation to the dichotomous mental health outcomes using binary logistic regression analysis. Because consuming energy drinks less than once a week and eating breakfast every day were considered to be the healthiest dietary practices, the frequent breakfast/infrequent energy drinks condition was chosen as the comparison group. In addition to this, the odds ratios and 95% confidence intervals were compared across the other three conditions; if the confidence intervals were found not to overlap, then the difference between groups was considered to be significant.

All dietary, demographic, and lifestyle data were available from two cross-sections (spaced 6 months apart), although information relating to stress, anxiety, and depression were only collected at the latter. Though this meant that changes in mental health status could not be investigated, cross-lag analyses were conducted to determine whether breakfast and energy drink consumption (both in isolation and in combination) were predictive of stress, anxiety, and depression levels at 6-month follow-up. These analyses therefore utilized the same procedures as the cross-sectional analyses, except that in this case the predictor variables and covariates came from T1 rather than T2. All data analysis was conducted using IBM SPSS Statistics Version 20. For details of all predictor variables and covariates used in the multivariate analyses, see **Table 1**.

**Table 1:** Predictor variables and covariates entered into cross-sectional and cross-lag binary logistic regression analyses upon the outcomes of stress, anxiety, and depression.

	Variable	Type	Description
Predictor	Breakfast	Categorical	Frequency of breakfast consumption derived from DABS single item: every day vs. not every day
Variables	Energy drinks	Categorical	Frequency of energy drink consumption derived from DABS single item: less than once a week vs. once a week or more
	Breakfast and energy drink combinations	Categorical	All four combinations of frequent/infrequent breakfast and energy drink consumption derived from the above two variables
Dietary	Junk food	Continuous	DABS subscale score
Covariates	Healthy foods	Continuous	DABS subscale score
	Weekly caffeine from energy drinks^∗^	Continuous	Caffeine consumed specifically from energy drinks; derived from DABS single item
	Weekly caffeine from cola	Continuous	Caffeine consumed specifically from cola; derived from DABS single item
	Weekly caffeine from tea	Continuous	Caffeine consumed specifically from tea; derived from DABS single item
	Weekly caffeine from coffee	Continuous	Caffeine consumed specifically from coffee; derived from DABS single item
Demographic	School	Categorical	School attended (Academy 1, Academy 2, or Academy 3)
Covariates	School year	Categorical	Year group attended (Year 7, 8, 9, 10, or 11)
	Sex	Categorical	Male or female
	Special educational needs (SEN)	Categorical	Presence or absence of a SEN status
	Free school meals (FSM)	Categorical	Eligibility or ineligibility to receive FSM
Lifestyle	Sleep	Continuous	Average number of sleep hours achieved per night
Covariates	Exercise	Continuous	Factor score derived from three single items (frequency of mild, moderate, and vigorous exercise)
	School attendance	Continuous	School attendance percentage

*Predictor variables were entered individually (i.e., in the absence of other predictor variables). All covariates were entered into each analysis except where noted. ^∗^Caffeine from energy drinks was only entered as a covariate when the predictor variable was breakfast consumption.*

## Results

Descriptive statistics relating to variance in demography and lifestyle for this sample have been reported in Richards and Smith (2015a). In addition, frequency data for stress, anxiety, and depression are available in Richards and Smith (2015b). This latter study observed a large amount of variability in mental health status. Though the most commonly reported frequency for experiencing depression was ‘not at all’ (36.1%), anxiety was most commonly reported as occurring ‘rarely’ (38.2%), and stress was most commonly reported as occurring ‘sometimes’ (37.4%). Mental health problems that were experienced ‘very frequently’ were relatively uncommon (stress = 8.5%, anxiety = 4.6%, depression = 4.8%).

### Associations Between Covariates, Predictor Variables, and Outcomes

#### Associations Between Covariates and Predictor Variables

In order to assess relationships between the predictor variables and covariates, the single item DABS questions for frequency of consumption of breakfast and energy drinks were recoded into dichotomous variables; breakfast was coded as ‘every day’ vs. ‘not every day’ (answer 5 vs. answers 1, 2, 3, and 4), and energy drinks was coded as ‘once a week or more’ vs. ‘less than once a week’ (answers 3, 4, and 5 vs. answers 1 and 2). Relatively equal numbers were present for participants who ate breakfast every day (1108; 48%) and those who did not eat breakfast every day (1198; 52%); for energy drinks, the majority of participants used the products less than once per week (1728; 75.4%), with relatively few using them once a week or more (563; 24.6%). These variables were then investigated in relation to categorical covariates using Chi-square tests, and in relation to continuous covariates using between-subjects *t*-tests.

Those who consumed breakfast every day were more likely to be male, χ^2^(1, *N* = 2092) = 39.749, *p* < 0.001, to be ineligible for FSM, χ^2^(1, *N* = 2251) = 11.701, *p* = 0.001, and to not have a SEN status, though the latter effect was only marginally significant, χ^2^(1, *N* = 2274) = 3.058, *p* = 0.08. Although breakfast consumption did not differ across the three schools investigated, χ^2^(2, *N* = 2306) = 1.264, *p* = 0.531, a significant effect was observed regarding the year group attended, χ^2^(4, *N* = 2250) = 21.007, *p* < 0.001. This reflected a significant linear trend in which the likelihood of eating breakfast every day decreased throughout secondary education, χ^2^(1, *N* = 2250) = 18.176, *p* < 0.001.

Compared to those who did not eat breakfast every day, those who did slept for longer, *t*(2190.355) = 12.041, *p* < 0.001, exercised more frequently, *t*(2139) = 2.57, *p* = 0.01, and achieved higher school attendance, *t*(2232.478) = 7.019, *p* < 0.001. Though not associated with Junk Food, *t*(2200) = 0.993, *p* = 0.321, those who ate breakfast every day achieved higher Healthy Foods scores, *t*(2217) = 5.837, *p* < 0.001, and consumed less caffeine from energy drinks, *t*(2093.156) = –6.819, *p* < 0.001, cola, *t*(2243.738) = –4.991, *p* < 0.001, and coffee, *t*(2206.026) = –2.927, *p* = 0.003. However, no difference was observed regarding the amount of caffeine consumed from tea, *t*(2265) = 0.19, *p* = 0.85.

Drinking energy drinks once a week or more was significantly associated with being male, χ^2^(1, *N* = 2078) = 56.833, *p* < 0.001, having a SEN status, χ^2^(1, *N* = 2259) = 42.149, *p* < 0.001, and being eligible for FSM, χ^2^(1, *N* = 2237) = 35.713, *p* < 0.001. School year was also associated, χ^2^(4, *N* = 2236) = 29.816, *p* < 0.001, reflecting a significant linear relationship in which energy drink consumption increased throughout secondary education, χ^2^(1, *N* = 2236) = 25.957, *p* < 0.001. Although more frequent energy drink users than expected came from Academy 3 (adjusted residual = 2), no overall significant difference between the schools was observed, χ^2^(2, *N* = 2291) = 4.563, *p* = 0.102.

Compared to those who consumed energy drinks less than once a week, those who consumed them once a week or more slept for fewer hours per night, *t*(770.321) = –8.547, *p* < 0.001, achieved lower school attendance, *t*(815.279) = –6.604, *p* < 0.001, and exercised more frequently, *t*(2130) = 1.933, *p* = 0.053 (though the latter effect was only marginally significant). In addition to this, frequent energy drink users achieved higher Junk Food scores, *t*(2196) = 8.619, *p* < 0.001, lower Healthy Foods scores, *t*(2206) = –4.675, *p* < 0.001, and consumed more caffeine from energy drinks, *t*(563.169) = 23.054, *p* < 0.001, cola, *t*(699.209) = 7.751, *p* < 0.001, and coffee, *t*(656.091) = 5.949, *p* < 0.001. As with breakfast consumption, there was no association between energy drink use and the amount of caffeine consumed from tea, *t*(2253) = 0.448, *p* = 0.654.

#### Associations Between Covariates and Outcome Variables

The single item measures of stress, anxiety, and depression were recoded into dichotomous variables, with those who answered with 1 or 2 (‘never’ or ‘rarely’ experienced stress, anxiety, or depression) being placed into the above average mental health group, and those who answered with 3, 4, or 5 (‘sometimes,’ ‘frequently,’ or ‘very frequently’ experienced stress, anxiety or depression) comprising the below average mental health group.

Compared to the low stress group, the high stress group achieved marginally lower Junk Food scores, *t*(2158) = 1.949, *p* = 0.051, though there was no difference regarding Healthy Foods, *t*(2176) = 1.072, *p* = 0.284. The low anxiety group on the other hand achieved higher Healthy Foods scores, *t*(2167) = 2.373, *p* = 0.018, but there was no difference for Junk Food, *t*(1902.825) = 1.321, *p* = 0.187. There was also a marginally significant trend for those in the low depression group to consume more Junk Food, *t*(1449.921) = 1.796, *p* = 0.073, though no effect on Healthy Foods was observed, *t*(2165) = 0.591, *p* = 0.554. Richards and Smith (2015a) have already reported associations between the dichotomous stress, anxiety, and depression outcomes and caffeine consumed from energy drinks, cola, coffee, and tea in this sample. At the univariate level the study found caffeine intake from coffee to be positively associated with stress, anxiety, and depression (though the effects appeared to be due to coffee consumption being a strong predictor of overall caffeine intake), and caffeine consumed from cola to be associated with low stress (potentially reflecting a coping strategy).

Associations between the dichotomous stress, anxiety, and depression variables and demographic and lifestyle covariates used in the current study have been reported by Richards and Smith (2015b). At the univariate level, their analysis found female gender, low sleep hours, infrequent exercise, and low school attendance to be predictive of high levels of stress, anxiety, and depression. Although no differences were detected regarding the schools attended, stress, anxiety, and depression were each found to increase throughout secondary education. In addition to this, those with a SEN status were more likely to report high levels of depression, and those eligible for FSM were more likely to report low anxiety.

### Cross-Sectional Associations Between Breakfast and Energy Drink Consumption, and Stress, Anxiety, and Depression

Breakfast (every day vs. not every day) and energy drinks (once a week or more vs. less than once a week) were each entered separately (i.e., they were not entered into the same model) into binary logistic regression analyses upon the dichotomous stress, anxiety, and depression outcomes, along with dietary, demographic, and lifestyle covariates. Not eating breakfast every day was associated with high stress, OR = 1.324, 95% CI [1.064, 1.647], *p* = 0.012, anxiety, OR = 1.35, 95% CI [1.088, 1.674], *p* = 0.006, and depression, OR = 1.515, 95% CI [1.212, 1.894], *p* < 0.001, though no significant effects were observed relating to energy drinks: stress, OR = 1.13, 95% CI [0.863, 1.479], *p* = 0.375; anxiety, OR = 0.94, 95% CI [0.72, 1.227], *p* = 0.648; depression, OR = 1.131, 95% CI [0.863, 1.482], *p* = 0.373.

In order to investigate their potential combined effects, the dichotomous breakfast and energy drink consumption variables were combined into the following four groups: (1) breakfast every day/energy drinks less than once a week (*N* = 919; 40.1%), (2) breakfast every day/energy drinks once a week or more (*N* = 184; 8%), (3) breakfast not every day/energy drinks less than once a week (*N* = 809; 35.3%), (4) breakfast not every day/energy drinks once a week or more (*N* = 378; 16.5%). For ease of reporting, the frequency of breakfast and energy drink consumption will both henceforth be referred to as ‘frequent’ or ‘infrequent’. Binary logistic regression analyses were conducted, and the frequent breakfast/infrequent energy drinks group was set as the control. The same covariates as entered in the analyses of energy drink consumption in isolation were once again used here.

**FIGURE 1:**
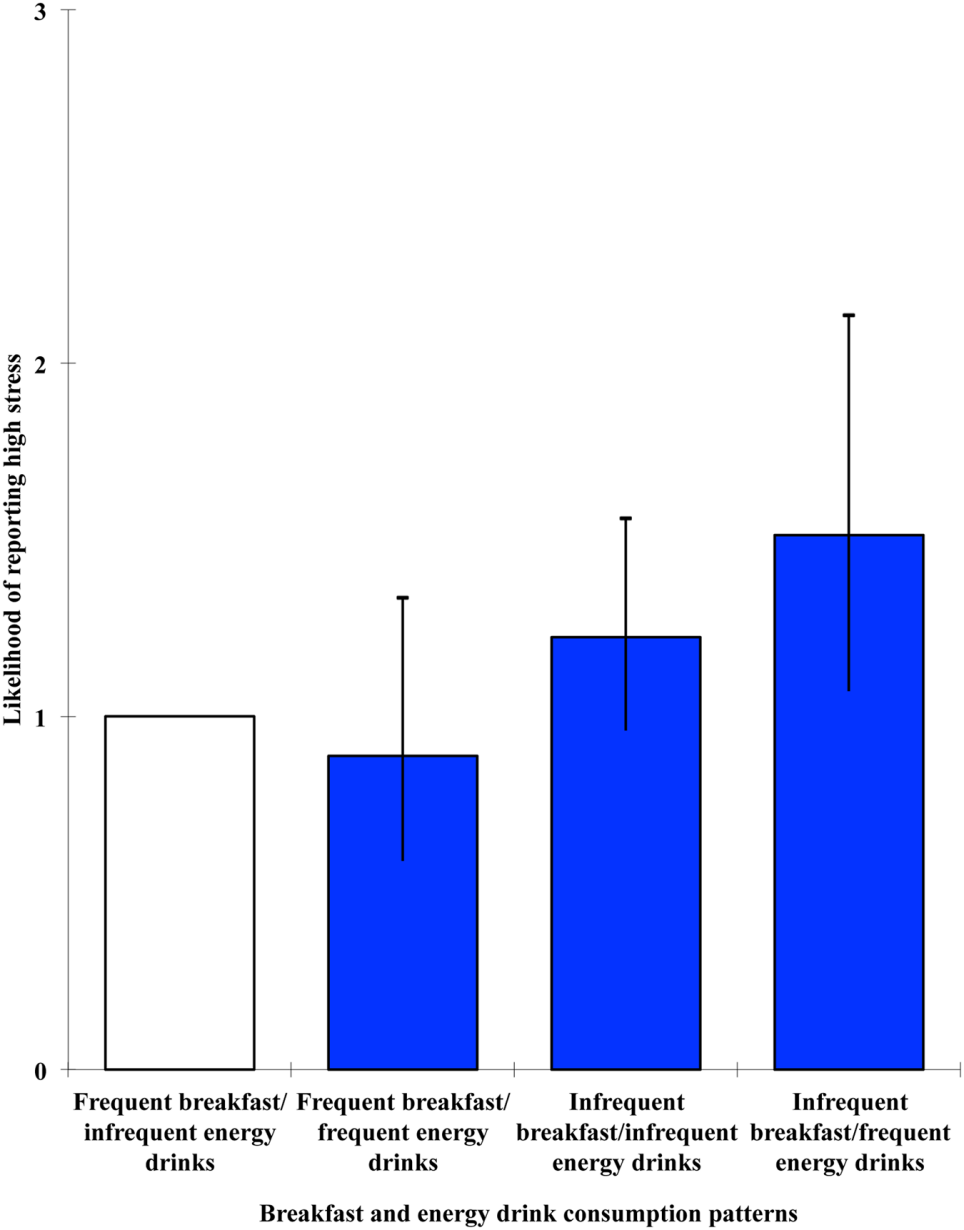
**Likelihood of reporting high stress as a function of breakfast and energy drink consumption combinations**.

**FIGURE 2:**
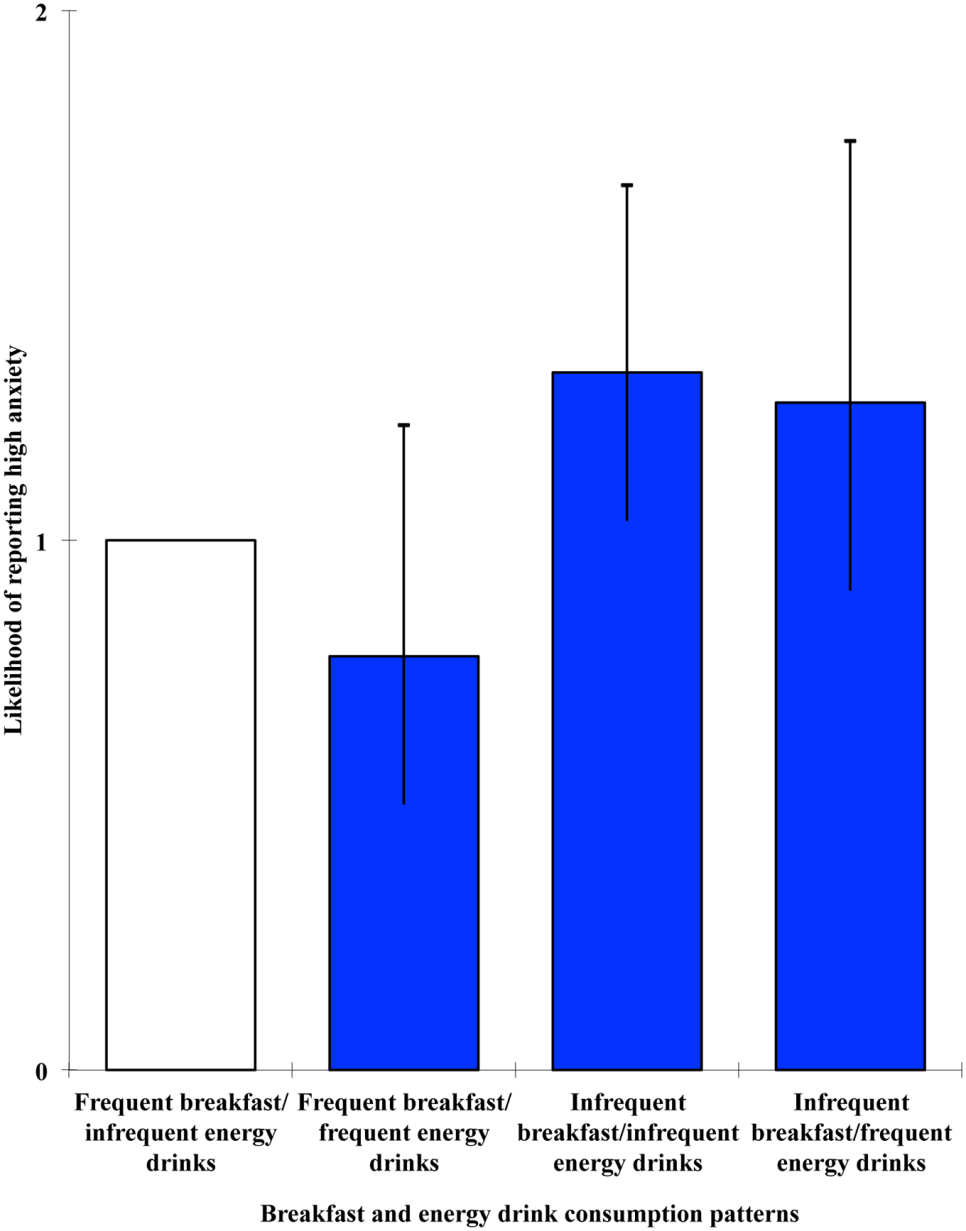
**Likelihood of reporting high anxiety as a function of breakfast and energy drink consumption combinations**.

**FIGURE 3:**
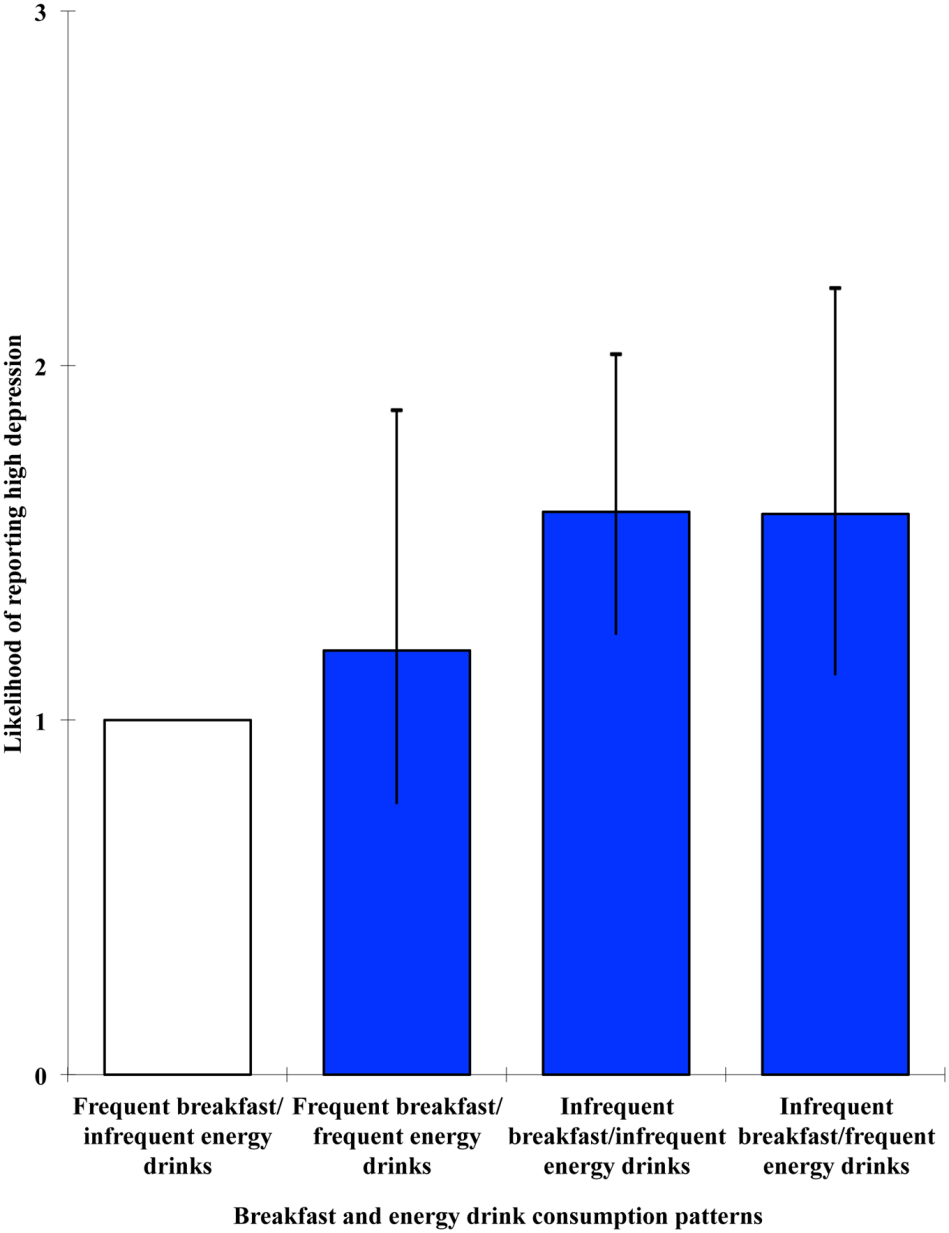
**Likelihood of reporting high depression as a function of breakfast and energy drink consumption combinations**.

The effect of breakfast and energy drinks groups was significant in relation to each of the outcome variables: stress, Wald = 8.228, *p* = 0.042; anxiety, Wald = 8.791, *p* = 0.032; depression, Wald = 15.17, *p* = 0.002. High stress levels were significantly associated with being a member of the infrequent breakfast/frequent energy drinks condition, OR = 1.513, 95% CI [1.072, 2.135], *p* = 0.018, whereas high anxiety was associated with the infrequent breakfast/infrequent energy drinks condition, OR = 1.317, 95% CI [1.037, 1.671], *p* = 0.024. High levels of depression, on the other hand, were associated with both groups that did not consume breakfast every day: infrequent breakfast/infrequent energy drinks, OR = 1.587, 95% CI [1.24, 2.032], *p* < 0.001; infrequent breakfast/frequent energy drinks, OR = 1.581, 95% CI [1.127, 2.218], *p* = 0.008. For visual representations of the odds ratios and 95% confidence intervals for the cross-sectional multivariate level combined effects of breakfast and energy drinks on stress, anxiety and depression, see **Figures 1–3**, respectively.

### Cross-Lag Associations Between Breakfast and Energy Drink Consumption, and Stress, Anxiety, and Depression

In order to determine whether the frequency of breakfast and energy drink consumption was predictive of stress, anxiety, and depression at 6-month follow-up, a cross-lag analysis was conducted. For this, the same predictor variables and covariates as used in the cross-sectional analysis were used, except that they came from T1 rather than T2. Breakfast consumption at T1 was not associated with any of the outcome measures at T2: stress, OR = 1.2, 95% CI [0.936, 1.538], *p* = 0.15; anxiety, OR = 1.061, 95% CI [0.833, 1.352], *p* = 0.632; depression, OR = 1.16, 95% CI [0.907, 1.483], *p* = 0.237. This was also true for energy drink consumption: stress, OR = 0.92, 95% CI [0.695, 1.219], *p* = 0.562; anxiety, OR = 0.856, 95% CI [0.648, 1.131], *p* = 0.274; depression, OR = 1.001, 95% CI [0.756, 1.324], *p* = 0.996. Furthermore, the combined effect of breakfast and energy drinks at T1 was not predictive of future levels of stress, Wald = 2.018, *p* = 0.569, anxiety, Wald = 3.873, *p* = 0.275, or depression, Wald = 2.067, *p* = 0.559.

## Discussion

Due to reports in the literature associating breakfast omission and energy drink consumption with undesirable outcomes, the current study aimed to investigate these dietary variables in relation to self-reported stress, anxiety, and depression in a large cohort of secondary school children from the South West of England. In addition to this, as Richards et al. (2015a) reported that a combination of breakfast omission and energy drink use was predictive of the acute occurrence of behavioral sanctions, the combined effects of these variables were also investigated. It was hypothesized that a combination of breakfast omission and frequent energy drink consumption would be the strongest predictor of high stress, anxiety, and depression levels.

### Effects of Breakfast and Energy Drinks on Stress, Anxiety, and Depression

At the cross-sectional level, eating breakfast every day was found to be predictive of low stress, anxiety, and depression. These effects generally reflected those already reported in the literature (e.g., Smith, 1998; O’Sullivan et al., 2009). Energy drink consumption on the other hand, was not associated with any of the mental health outcomes. This was somewhat surprising considering that a number of studies have previously reported positive relationships between energy drink use and stress (Hofmeister et al., 2010; Pettit and DeBarr, 2011), anxiety (Hofmeister et al., 2010; Stasio et al., 2011; Trapp et al., 2014), and depression (Azagba et al., 2014). However, it should also be noted that a number of such studies (e.g., Hofmeister et al., 2010; Arria et al., 2011; Trapp et al., 2014) have provided mixed results.

Combined effects of breakfast and energy drinks were observed in relation to stress, anxiety, and depression. In line with the hypothesis, high stress was associated with the infrequent breakfast/frequent energy drinks condition. However, high anxiety was associated with the infrequent breakfast/infrequent energy drinks condition, and high depression was associated with both groups that did not consume breakfast every day. Taken together, these findings suggest that breakfast omission is consistently associated with negative mental health outcomes, and that such effects can generally be observed in those who frequently consume energy drinks as well as those who do not. However, when investigating the predictive value of breakfast omission and energy drink consumption, both in isolation and in combination, on stress, anxiety, and depression at 6-month follow-up, no significant effects emerged. This therefore suggests that the relationships observed at the cross-sectional level are unlikely to be casual in nature and there may be bi-directional mechanisms involved, with mental health also influencing whether or not breakfast is consumed.

### Methodological Limitations and Directions for Future Research

Although the current study has a number of strengths (e.g., large sample size, multivariate approach to analysis), several limitations should be acknowledged. Considering both the minimum and average age of the participants, it is possible that some will not have fully understood the difference between each of the mental health problems in a way that they could tell to what degree they had suffered them over the previous 6 months. Due to this, the results of the study should be interpreted tentatively. In addition to this, the sample population was not entirely representative of the schools from which it came (see Richards and Smith, 2015a), and was also somewhat homogeneous, being comprised mainly of White schoolchildren from a specific geographical location. Though the methodology used attempted to control for these factors statistically, future research should aim to investigate similar dietary effects in more representative populations in order to increase the generalizability of findings.

Single items were also used to measure the mental health outcomes. These single items have been shown to be highly correlated with full length scales in studies of working age populations and university students. However, we do not know whether this is also true of adolescents and it may be the case that use of single items in this group requires examples of what is meant by stress, anxiety, and depression. Another issue incurred by the current study is that information relating to mental health was only collected at one time-point. This meant that, although cross-lag analyses could be conducted, changes in stress, anxiety, and depression could not be investigated. The cross-sectional analyses presented therefore incur the problem of reverse causation, and so, cause and effect cannot be determined. For instance, though breakfast omission may increase stress, anxiety, and depression, suffering from such conditions may also lead to sleep disturbances, reducing the likelihood of eating breakfast the following morning. Though the cross-lag analyses provided evidence to suggest that associations between the dietary variables examined in this study and stress, anxiety, and depression were unlikely to be causal, they were themselves confounded by the fact that the two cross-sections were collected only 6 months apart. There is therefore a need for further longitudinal and intervention studies to better determine the nature of the effects so far observed.

## Conclusion

The current study has provided evidence to suggest that high stress, anxiety, and depression levels in adolescents are associated with breakfast omission, and that such associations with energy drink use may be more variable. However, all significant effects observed were at the cross-sectional level, with cross-lag analyses finding these dietary patterns not to be predictive of mental health outcomes at 6-month follow-up. Nevertheless, given that the findings may still have implications regarding school policy, it is considered important that the combined effects of breakfast and energy drinks are investigated further, and in relation to other important outcomes such as academic attainment and school attendance.

## Author Contributions

AS conceived and designed the study, and GR analyzed and interpreted the data. GR also drafted the original manuscript, and AP revised it for important intellectual content. Both authors approved the final version of the manuscript for publication, and agree to be accountable for all aspects of the work in ensuring that questions related to accuracy and integrity are appropriately investigated and resolved.

## Conflict of Interest Statement

The authors declare that the research was conducted in the absence of any commercial or financial relationships that could be construed as a potential conflict of interest.
